# A review of malaria transmission dynamics in forest ecosystems

**DOI:** 10.1186/1756-3305-7-265

**Published:** 2014-06-09

**Authors:** Narayani Prasad Kar, Ashwani Kumar, Om P Singh, Jane M Carlton, Nutan Nanda

**Affiliations:** 1 Indian Council of Medical Research, National Institute of Malaria Research, Sector-8, Dwarka, New Delhi 110077, India; 2 National Institute of Malaria Research, DHS Building, Campal, Panaji, Field Unit Goa-403001, India; 3 Department of Biology, New York University, 12 Waverly Place, New York, NY 10009, U.S.A

**Keywords:** Forest malaria, Transmission dynamics, Deforestation, Vector behavior, Socio-economic factors, Tribal communities

## Abstract

Malaria continues to be a major health problem in more than 100 endemic countries located primarily in tropical and sub-tropical regions around the world. Malaria transmission is a dynamic process and involves many interlinked factors, from uncontrollable natural environmental conditions to man-made disturbances to nature. Almost half of the population at risk of malaria lives in forest areas. Forests are hot beds of malaria transmission as they provide conditions such as vegetation cover, temperature, rainfall and humidity conditions that are conducive to distribution and survival of malaria vectors. Forests often lack infrastructure and harbor tribes with distinct genetic traits, socio-cultural beliefs and practices that greatly influence malaria transmission dynamics. Here we summarize the various topographical, entomological, parasitological, human ecological and socio-economic factors, which are crucial and shape malaria transmission in forested areas. An in-depth understanding and synthesis of the intricate relationship of these parameters in achieving better malaria control in various types of forest ecosystems is emphasized.

## Background

### Stratification of global malaria

Malaria is an infectious disease caused by parasites belonging to the genus *Plasmodium*. It is endemic in 104 tropical and subtropical countries, comprising half of the world’s population (3.4 billion people) [1], of which 2.57 billion are at risk for *P. falciparum*[2], and 2.5 billion for *P. vivax*[3]. *P. malariae* and *P. ovale* contribute a very small proportion of malaria infections but the population at risk of *P. malariae* is distributed all over sub-Saharan Africa, most parts of Southeast Asia, western Pacific islands, and Amazonian Basin [4,5]. *P. ovale* is prevalent in Africa [5], and it is also reported from Asia-Pacific regions [6]*. P. knowlesi,* the fifth human parasite [7] is essentially a primate malaria species that is being reported from remote forested areas of Southeast Asian countries [8-11].

#### Forest malaria

##### Definition of “forest”

The ‘forest ecotype’ is defined by UNESCO as terrain with a tree canopy cover of more than 10% and an area of more than 0.5 hectares, including natural forests and plantations [12] with a minimum tree height of 5 m, including coffee, rubber, cork oak, and fruit tree plantations, wind break and shelter belts more than 20 m width [13]. Forest vegetation is categorized as rain forest, deciduous forest, scrub forest, highland rain forest, and highland alpine forest [14]. The former three are usually distributed in low to mid latitude and the last two are part of the high altitude biome.

##### Impact of forest malaria

Forest ecosystems are well known to support transmission of malaria, significantly contributing to the global disease burden. A global assessment reports that “closed forests within areas of malaria risk cover approximately 4.8 million km^2”^[12]. Almost half the malaria risk is estimated to occur among people living in forested areas (1.4 billion) accounting for 11.7, 18.7, 35.1 and 70.1 million population respectively from 1.5 million km^2^ in the Amazon region, 1.4 million km^2^ in Central Africa, 1.2 million km^2^ in the Western Pacific, and 0.7 million km^2^ in South–East Asia [15,16]. Corresponding forest areas containing these malaria risk zones are 11.16 million to 15.71 million km^2^, 6.53 million– 7.80 million km^2^, 1.93 million– 5.19 million km^2^, 2.70 million–2.72 million km^2^[12,15,16]. Controlling malaria in these forested regions of the world has been a major challenge [17].

### Summarizing hidden risks of malaria in forests

Most studies of forest malaria are focused on local factors associated with malaria transmission. These include distance from forest, impact of deforestation and reforestation, effect of forest on microclimate, vector bionomics, *Plasmodium* species survival, and human activities in forests. In this review we analyze the underlying factors influencing transmission of malaria in forests worldwide. Mosquito vectors vary according to forest locality and their behavior changes with the forest micro-climate [18], human population, and their social behaviors [19,20]. Forest communities are generally tribal and cope with poor infrastructure. Certain practices like slash and burn cultivation, overnight stays within forests in order to collect forest produce, hunting, wide open household construction, and cattle ranching, increase vulnerability to malaria. It can be challenging to educate forest communities about malaria control, and without their cooperation it is difficult to control malaria [21]. Now, worldwide malaria communities are aiming at malaria eradication/elimination [22,23], a proposition, which is impractical without prevention of re-introduction/re-emergence from hidden foci/uncontrolled forest malaria [22,24]. Malaria had declined during the previous eradication era in many regions of the world, some of which subsequently experienced resurgence and suffered from its consequences [23,25-27]. The problem of malaria in forests is compounded by hidden reservoirs of malaria infections that are not fully addressed [28,29]. The origin and evolution of drug and insecticide resistance are often found associated with the forest and near forest areas [24,30-32]. In addition to existing asymptomatic infections, the presence of primate malaria parasites and their zoonotic vectors might pose additional challenges to human health in the forest and forest fringe areas [33-35], where malaria surveillance is generally poor [33,36]. Occasional focal outbreaks (unstable transmission) might occur when malaria transmission extends from the forest shade (nidus) to peri-urban and urban areas [37], where much higher density of human population and presence of vectors could fuel large epidemics.

### Key factors which make forest different from other ecosystems

The major factors, that differentiate forest from other ecosystems in relation to malaria transmission dynamics, are the influence of forest on temperature buffering, rainfall [38], humidity [39-41], tree canopy [42], flora, fauna [43], high organic content in breeding pools [44], and lack of infrastructure [45]. It is difficult to develop infrastructure in forests due to their uneven land forms, presence of streams, and dense vegetation. Additionally, poor communication hinders malaria control activities particularly during the rainy season [21,45]. Furthermore, forests with hilly land forms are more malariogenic, as their slopes form small rapid streams that facilitate breeding of efficient malaria vectors [46]. Forest influences vector distribution and bionomics, and also the distribution of the malaria parasites. Forested areas are primarily inhabited by tribes [47,48], whose illiteracy and strong beliefs in age old traditions and practices and a fear of outside world leads to reliance on indigenous treatment for malaria [49].

These factors are discussed in detail in the following sections.

## Review

### Influence of topographic parameters in forested regions

Topographic factors influence malaria transmission dynamics in forests differently compared to other regions [38,40,50-52] as forests harbor forest adapted malaria vectors [53,54] which respond differentially to these parameters due to their genetic makeup and presence of forest influences their ecology to a great extent in comparison to less or no forest areas [18,28,52,55].

### Temperature, rainfall, and humidity

In forested highlands of East Africa, average temperature in the previous month and rainfall in the previous two months have shown a linear-quadratic relationship with *Anopheles gambiae* density [51]. Another study in the same region showed that the ratio of rainfall over precipitation/potential evapo-transpiration was the driving force for *An. gambiae* and *An. arabiensis* population increase [40]. The same vector studied in The Gambia showed rapid population increase towards the end of the dry season, and maximally after onset of rains when humidity increases [56]. Generally trees in the forests add moisture in the air by transpiration and help in lowering temperature, thus increasing precipitation. The moist environment and breeding sites created by rainfall increase vector population, their longevity and hence increase malaria transmission [19].

### Vegetation

Vegetation near human habitation increases the population of forest malaria vectors and thus increases malaria transmission [57-59]. Villages with more broadleaf forests, and wetland vegetation in Belize and in forested villages of Bangladesh have higher malaria rates [60,61] due to effective density of forest vectors [61]. Forest vectors usually prefer tree canopy coverage [42,62] and are known to take shelter in tree holes [63,64]. Forest flora and sugar availability have also been shown to be crucial determinants of vectorial capacity. The availability of plant sugar increased egg numbers [43,65] and survival potential of *An. gambiae* beyond ages at which they are old enough to transmit malaria [66]. In addition, leaves falling into larval habitats assure sustainable micro-climatic conditions and food for larvae, which favor vectors like *An. dirus* in South-East Asia [52].

### Bodies of water

Mosquitoes mature in bodies of water (their larval habitat) and disperse according to their flight range. For example *An. gambiae* and *An. funestus* populations were observed decreasing with increasing distance from the Yala river in Kenya [42]. Even a small change in the distance from bodies of water can influence malaria transmission [19,67]. *Anopheles fluviatilis*[68], *Anopheles maculatus*[68,69] and *An. minimus*[52,68,69] are prevalent near streams of water in forested areas having cooler climate and tree canopy [70], but *An. dirus* larvae grow well in small, clear and stagnant bodies of water in forested areas of Asia [52,68]. In Africa, *An. gambiae s.s* larvae grow better in bodies of water under dense forest canopy rather than sparse forest coverage [71]. Generally, larvae of forest vectors develop better in bodies of water under tree canopy where the water temperature is buffered and usually 3–3.5 degrees Celsius lower than that of sun-exposed bodies of water [71].

### Deforestation

Reduction of dense tree shade increases exposure of vector breeding sites and resting places to sunlight, hence altering vector habitats. Studies have shown preference for forest shade by, *An. dirus*[52,72], *An. fluviatilis*[72,73], *An. minimus*[72,74], and *An. funestus*[74,75], *An. darlingi*[72] and contrastingly, preference for sunlight is shown by some of the species of *An. gambiae*[72,75], and *An. maculatus*[68,69,76]. Changing density of anophelines due to deforestation has been reported worldwide, and its relation to niche width and sunlight preference were reviewed in meta-analysis/tabulation [72], and it was found that changes in anophelines density and malaria incidence varied by type of development, agriculture, and locality [72]. It was predicted that deforestation in central Africa and tropical America might increase malaria [12,77], whereas in Asia deforestation would result in reduction in malaria [78]. As predicted in the Sahara region, malaria incidence increased due to deforestation as a consequence of increased vector density of *An. gambiae* and *An. arabiensis*[72], and increase in *An. funestus* and *An. gambiae* population in Sub-Saharan Africa [72]. Similarly deforestation increased the population of the South American vectors *An. darlingi* and *An. aquasalis*[72], accompanied by increased malaria in Guyana and Amazonia [72]. The predicted reduction in malaria in deforested regions of Asia may be due to a decrease in forest-loving (halophobic) vectors like *An. dirus* in Thailand and *An. fluviatilis* in India [73,79]. However, malaria transmission was accelerated by *An. minimus* due to deforestation in Thailand and India [80], as well as *An. culicifacies* in Nepal and Sri Lanka [72], and *An. philippinensis, An. annularis,* and *An. varuna* in India [80]. A risk of increased malaria in response to deforestation exists if vectors like *An. darlingi* are present in a deforested habitat [81], as the biting rate of *An. darlingi* has been estimated to increase 278 times in the deforested regions [82]. Thus deforestation affects malaria transmission depending upon the vector diversity of a particular region.

#### Entomological parameters of forested regions

##### Impact of forest and forestation on vector abundance

The vector species in forest ecoregions reflect their preference or adaptability to the forest ecotype. Malaria transmission dynamics in forests may be the output of more than one vector [83]. Different vector species or sympatric sibling species may be present in a particular region whose populations fluctuate according to seasons [52,84]. In the dense, hilly forested Thai-Myanmar border region, more *An. dirus* were found at the start while more *An. baimaii* were found towards the middle of the wet season [52]. In the less forested southern part of Thailand, however, *An. cracens* dominated over *An. baimaii* at the beginning of the wet season though by the end of the wet season *An. scanloni* dominated *An. cracens*[52,84]. In the subtropical mountainous forest of northwestern Argentina, *An. argyritarsis* was more abundant than *An. pseudopunctipennis* and both the vectors attained their peaks during spring [85]. Among the species of the Gambiae Complex, *An. gambiae s.s.* is less adapted to hotter conditions than *An. arabiensis*[86,87], hence the former is more abundant in forest than desert in comparison with the latter species, as reflected by their spatial and temporal distribution in Africa [29,54,88-90]. During dry periods when the primary forest vector (*e.g., An. gambiae s.s.*) population decreases, the secondary vector (*e.g., An. arabiensis*) takes over the transmission of malaria.

Manmade forests including significantly large plantation areas or reforestation also cause habitat change and influence malaria vector abundance leading to changes in malaria transmission scenarios. For example malaria increased due to a coffee plantation in Thailand [91], palm plantations in Cameroon [92], Papua New Guinea [93] and Malaysia [94]; rubber plantation in Cameroon [95], Thailand [91] and orchard plantations in Thai-Myanmar and other South-East Asia regions [91,96,97]. Commercial plantations and reforestation, which increase human insurgence, increases man-vector encounter and malaria transmission in those areas [78,91,96,97].

### Behavior of forest-adapted vector forms

Some non-forest vectors have distinct forest forms that exhibit alteration in chromosomal banding pattern and show altered bionomics compared to their non-forest forms [53,54,98]. The variation in vector forms is accompanied by differences in vectorial capacity, biting habits and differential resistance to insecticides, thus influencing vector control strategies [99,100] in response to malaria transmission [98,101]. Obsomer *et al.*, 2007 in their review on *An. dirus* in Asian forested zones, emphasized forest environment, human behavior, and insecticide usage over vector genetics to account for the vector’s behavioral heterogeneity [52], which also supports the earlier views of Trung and co-workers [18,28]. They reviewed behavioral heterogeneity of anophelines in South-East Asia with reference to forest, hill, and other factors and found that early evening shift in the human biting rhythm of *An. dirus* A (*An. dirus*) and *An. minimus* A (*An. minimus* Theobald), and higher degree of exophagy are inversely related to distance from forests and hills [52]. The forest form of *An. gambiae* has shown stronger exophily in southern Sierra Leone whereas the Savannah form was mostly endophilic [98]. Daytime biting by *An. dirus* was also observed in the forest, where very little sunlight penetrates through the tree canopy [52,102].

Non-forest vectors showed altered bionomics in the forest. For example *An. culicifacies* which is mainly endophilic [103] but in dense forests of central India, it is reported mainly exophilic in nature [104]. Similarly *An. gambiae* was observed to be highly exophilic and anthropophagic in a forested region compared to a non-forested region of southern Sierra [55]. The highest anthropophagic index and sporozoite positivity was observed in the savanna forest region for all four major malaria vectors *An. gambiae, An. funestus, An. arabiensis,* and *An. moucheti* in Nigeria [105], and for *An. gambiae* in Southern Ethiopia [106] and Madagascar [107] in comparison with the less forested rainforest region of south-western Nigeria, where *An. arabiensis* was largely zoophagic, whereas *An. gambiae*, *An. melas* and *An. moucheti* remained predominantly anthropophagic [108]. The impact of forest/deforestation on vector populations, their bionomics and malaria incidence is summarized in Table 1.

**Table 1:** Impact of forest/deforestation on vector populations, their bionomics and malaria incidence

**Malaria vectors**	**Increase in anthropophagy**	**Increase in exophily/exophagy**	**Increase in vector population/malaria**
*An. gambiae*	Forested region of Southern Sierra [55]	Forested region of Southern Sierra [55]	Deforested region of Africa [72,109]
*An. arabiensis*	Savannah-forest region of Nigeria [105]	Forested region of Nigeria [110]	Deforested region of Africa [72]
*An. funestus*	Savannah-forest region of Nigeria [105]	No increase in exophagy reported from rain forest zone of Nigeria [110]	Deforested region of Africa [72]
*An. dirus*	Forested region of Thailand [52], & Vietnam [17]	Forested region of Vietnam [17,28]	Forested regions of Asia [72]
*An. fluviatilis*	Forested region of Orissa, India [111]	Forested region of Central India [104]	Forested region of Orissa, India [111]
*An. minimus*	Forested region of Kratie province, Cambodia [112] Deforested region of Central Vietnam [18]	Deforested region of Central Vietnam [18]	Deforested region of Asia [72,79,80]
*An. culicifacies*	Forested region of Orissa, India [111]	Forested region of Central India [104]	Deforested region of Asia [72]
*An. maculatus*	Forested region of Kratie province, Cambodia [112]	Forested region of Kratie province, Cambodia [112]	Deforested region of Asia [72]
*An. darlingi*	Deforested region of Peruvian Amazon [82]	Forested region of Brazil [113]	Deforested region of South America [72,82] and near forest area of South America [114]
*An. aquasalis*	Forested region of Guayana, Venezuela [67]	Deciduous dry forested region of Venezuela [67]	Deforested region of South America [72]

#### Parasitological factors in relation to vector and host

##### *Plasmodium* species distribution in forested regions

*Plasmodium* in humans is little influenced by forest factors, as its secondary lifecycle is completed in a homeotherm. However, the primary life cycle occurs in an ectothermic vector, which is very much influenced by environment. Intrinsic incubation period is triggered by unknown phenomena [115] but extrinsic incubation period is inversely related to temperature and also depends upon *Plasmodium* species and the vector [116]. *P. vivax* and *P. falciparum* have shorter extrinsic incubation periods and are also the most common human malaria parasites [117]. *P. vivax* can survive in places like the Central Andes where the weak vector *An. pseudopunctipennis* and fluctuating environmental conditions prevail and are compensated by the short extrinsic incubation period of *P. vivax*, long intrinsic incubation periods in the human liver [118], and by forest cover that increases the life expectancy of the vector [109]. *Plasmodium* species have evolved to fit local vectors, as observed in *P. falciparum* in rural Cameroon by shortening sporogony [119] as survival of the vector influences *Plasmodium* distribution [120]. An increase in vectorial capacity of *An. gambiae* was reported in deforested areas of Kenya as deforestation led to a decrease in duration of sporogony of *P. falciparum*[109]. *Plasmodium malariae, P. knowlesi* and *P. ovale* cases are rare and mainly confined to remote forested areas and are usually underreported as these are often misidentified [121-123].

### Risk of primate malaria to humans in forest regions

The presence of a non-human primate *Plasmodium* species in forest foci poses a constant risk of host switching to nearby human populations due to deforestation, with its associated insurgence of the human population [124]. *Anopheles* in subgenus Kerteszia (*An. Kerteszia cruzii, An. Kerteszia bellator*), are vectors of human and simian plasmodia in areas like Atlantic forest in South America [33,34]. It is possible that zoonosis may be present in such areas, as the parasites found in monkeys (*P. simium* and *P. brasilianum*) are genetically similar/related to human plasmodia (*P. vivax* and *P. malariae*) [35]. Such cases occur, albeit infrequently, inside the forest or on its edges whose identity may be confused with human Plasmodia being morphologically similar [33]. The simian *Plasmodium* could switch over to humans as appears to have occurred in the case of *P. cynomolgi* in India [125], *P. simium* in Brazil [126,127], *P. knowlesi* in Malaysia [20,124]. The presence of asymptomatic human reservoirs together with infected monkeys could maintain malaria transmission in a situation where routine malaria surveillance and control are difficult [33,36].

### Parasite reservoir and drug resistance

Stability of malaria transmission in endemic areas is also reported to be associated with asymptomatic *P. falciparum* and *P. vivax* reservoirs and hypnozoite reservoirs of *P. vivax*[128,129]*.* Asymptomatic malaria is often associated with forested regions of the world [130-133]. Due to the asymptomatic reservoir [131], ‘stable endemic malaria’ is maintained continuously in forested areas [134], and in non-forested areas with unstable environments the reservoir plays a very important role in bridging transmission seasons, as human reservoirs help the parasite in escaping from a harsh environment during which the vector population also decline below the critical transmissible level [134]. For example, intense perennial transmission through an asymptomatic malaria reservoir was reported in the forested riverine areas of Tanzania [131] and Gabon [130]. Asymptomatic patients having sub microscopic presence of parasites [129,135] act as a reservoir [132,136,137] and ready source of infection in vectors [136] and are one of the hidden obstacles in malaria control in forests.

Patients infected with drug-resistant *Plasmodium* carry transmissible gametocytes for a very long time, and act as reservoirs. *Plasmodium falciparum* and *P. vivax* are reported to be more often associated with drug resistance in forested areas where intensity of malaria transmission is higher and malaria control often neglected. For these reasons the forested Thai-Cambodia border is believed to be the “epicenter” for the origin of chloroquine resistance and evolution of multidrug resistance [24,30,31]. Recently, partial artemisinin-resistance in *P. falciparum* has emerged from the same area [24,32]. The faster dissemination of drug resistant strain is likely due to the presence of some of the very efficient forest vectors in South-East Asia such as *An. dirus* and *An. minimus;* these vectors showed 66% and 44% susceptibility to a drug-resistant strain of *P. falciparum* infected patient blood respectively [28,52], and the number of oocysts of the drug-resistant type was reportedly higher in *An. dirus*[52]*.* Chloroquine-resistant foci have also been found associated with *An. dirus* in South Asia [52,138]*.*

#### Human ecological and socioeconomic factors

##### Known malariogenic practices of forest natives

Deep forest areas are primarily inhabited by indigenous populations of ethnic minorities and tribals [47,48] that are in little touch with outer world and mostly dependent on the forest for sustenance [139]. Such communities are mostly illiterate, prone to superstitious beliefs, and poor at communicating with malaria control workers. Tribes inhabiting forests have conserved traditions and practices that have remarkable impacts on malaria transmission [17] thus these forest dwelling people are vulnerable to malaria [21]. Slash and burn is a functional element of forest area farming practices in many parts of the world [17,140] leading to deforestation and succession of halophilic vectors, hence changes in the malaria transmission pattern [17]. In this type of cultivation, 1–2 members of the family stay in a hut near the farm overnight, which in turn exposes them to malaria vectors [17,140]. For example, malariogenic conditions are created in the central mountainous and forested part of Vietnam where the Rag Lays tribes practice slash and burn [17,140], and also by commercial logging, and cattle ranching [141]. Certain ethnic groups in India ritually plaster their houses with fresh cow dung and mud that masks the insecticide on treated walls and render it ineffective for vector control [142]. Traditionally women cover more of their bodies and perhaps for this reason were found to be at lower malaria risk than men [17,61,143]. In many tribal cultures both men and women consume alcoholic beverages on a regular basis and this practice results in reduced self-protection against mosquito bites. Interestingly, it has been found that beer consumption increases human attractiveness to *An. gambiae* in experiments conducted in Burkina Faso [144].

##### Population migration in forest areas

Populations move within and out of the forest for a variety of reasons and this helps in malaria dissemination [145,146]. Daily short-distance movement is done for cattle grazing, hunting, fishing, farming, collecting forest products like leaf, wood, fruit, flower and honey, *etc.*[147]. Such movements increase the contact with efficient malaria vectors when night halt is done in the forest [145,148,149] and even in the daylight where vectors like *An. dirus* prevails [52,140]. Short-term movement of forest inhabitants to medium distances is observed during sowing and harvesting seasons [38]. Generally this type of movement draws malaria from forested areas to plain field areas [145]. For example, increase in movement of people both within the highlands of New Guinea and also between holo and hyper-endemic lowland areas and the highlands increased malaria spread [150]. Non-forest populations also visit forest areas for animal grazing and wood collection [151]. Refugees have been settled and many resettlement programs have been launched in forest areas, for example, in India, Bangladeshi refugees were shifted to the forested Chittagong hill district and the forested area of the Orissa–Chhattisgarh border near Bastar under ‘Dandakaranya’ project. The refugees contracted malaria from native tribes, which led to epidemics in Bastar [152,153]. According to Lindsay *et al.*, “Many of the first European settlers in Africa who sought refuge from the heat and diseases of the plains by moving to the cool and salubrious highlands also carried malaria with them” [154].

##### Poor infrastructure and communication

Forest inhabitants usually construct houses with mud, and infection increased among those living in muddy or poorly constructed houses near vector breeding places in Egypt [155], Ethiopia [156] and Kenya [42]. *An. minimus* A in central Vietnam exhibits a high anthropophilic and endophagic ratio, most likely influenced by the largely open houses with incomplete walls that allow it to easily detect human stimuli and enter into houses [157].

Health infrastructure and surveillance are neglected in remote forest areas [158] and it becomes impractical in the rainy season where road infrastructure is poor or absent [159]. Unfortunately, the rainy season is also the peak transmission period in most malarious areas [160] when adequate surveillance is required as patients find it difficult to access health facilities due to climatic and communication problems [21,45]. Thus the indigenous population generally relies on the health practices of local faith healers and/or quacks [161-163]. Modern health infrastructure among sparsely distributed forest settlements is far from adequate [139,164]. It is reported that treatment seeking behavior is inversely related to the distance from a health facility and communication problems [21,165,166].

##### People’s conceptions and cooperation

Different perceptions about malaria have been reported among tribal groups in different parts of the world. Certain tribes believe that malaria is caused by spirits, angry deities, black magic, or consider it a self-limiting fever in countries like India [167] and southwest Ethiopia [165]. Low cost treatment with traditional medicines, good accessibility and good communication with quacks are preferred most in remote forest areas far from government health centers as reported in rural Ethiopia [168]. In the forest areas health seeking is directly related to culture, faith and affordability of the health care [21,165]. It is observed that “health services may be underutilized and several health care instructions may be ineffective or ignored in traditional and transitional societies where people’s ideas and behavioral patterns conflict with the knowledge being passed to them” [161]. Generally poverty is the next most important factor in accessing a distant healthcare facility besides illiteracy, superstition, and cultural faith among the indigenous populations of most forest regions in Bangladesh [143]. Poor forest inhabitants do not own a bed net primarily due to a lack of availability of affordable nets in spite of the fact that they know the benefits of nets and would want to use them [169,170]. Hence not having bed nets, poor people cover their body and face with blankets, burn wood and shrubs to ward off mosquitoes, and due to lack of affordable modern medical facility they practice traditional remedies [171]. Surveillance is poor in remote forested areas [172] and presumptive treatment using antimalarials in low doses is taken for all sorts of fever [173], accelerating the development of drug resistance [174,175]. Thus the origin and evolution of drug resistance was reported first in forested remote areas like Cambodia and the Thai-Myanmar border areas [176].

##### Suggestions for overcoming major challenges to curbing malaria transmission in forest ecosystems

Forests escape malaria control efforts mainly due to inadequate roads and poor communication [21]. Communication infrastructure development should be the first priority as this will open new ways and opportunity to the inhabitants and boost their socio-economic status. Establishing and development of accessible health facilities and making them available to inhabitants by reducing gaps between locals and health providers can improve the situation. Social awareness for malaria control and involvement of traditional health providers and NGOs may help in filling the gaps in backward forested areas [21,162].

Malaria transmission respects no political boundaries and in forested borders people often migrate across the borders and carry malaria as seen in Thai-Myanmar border. The population migrations in forested regions are due to various reasons but mainly due to availability of work [49,97]. Military camps, radar stations, police, and other armed forces camps and big development projects like road and other infrastructure development, mining, agricultural activities like tea, rubber, coffee plantation and construction of dams employ large numbers of migrant workers. These people acquire malaria easily from natives [152]. The vulnerable migrants and refugees need to be screened and treated for malaria promptly. The military and other camps in forested region are required to put large efforts to fight against malaria together with the local people.

Tribals in forest areas, often hidden from outer world, are generally conservative and reluctant in treatment seeking. Social inhibitions, ignorance, superstitions, and negligence promote tolerance in symptomatic or asymptomatic malaria carriers, who do not seek treatment themselves and act as reservoirs of malaria parasites [21,143]. Mass screening may be useful in order to assess malaria sero-positivity among communities where asymptomatic malaria prevails and people are less prompt in seeking treatment [23]. Antigen based species specific rapid diagnostic test kits should be used in forested areas for instant on-site detection and treatment. Primitive nomadic tribes need to be accessed by the healthcare providers and should be given place in social structure and are required to be encouraged to use healthcare and other facilities.

Forests are reported to be the epicenter of drug resistance spread and low attainment rates in malaria control. Therefore, strictly controlled administration of antimalarials with periodic assessment of drug resistance status is suggested in forested areas. Molecular markers associated with antimalarial resistance need to be evaluated in the high transmission forested areas and can help in saving the valuable antimalarials for posterity [176].

Deforestation and climate change can conspire with other factors of forest-malaria to cause a boom in vector species and increases in their vectorial dimensions [20,82]. Ecological succession of malaria vectors attributable to climate and ecological changes needs to be explored and frequent inspection of abundance of vector species and an update on their distribution pattern, bionomics and behavioral changes in forested areas are justified for effective vector control measures.

## Conclusion

**Figure 1:**
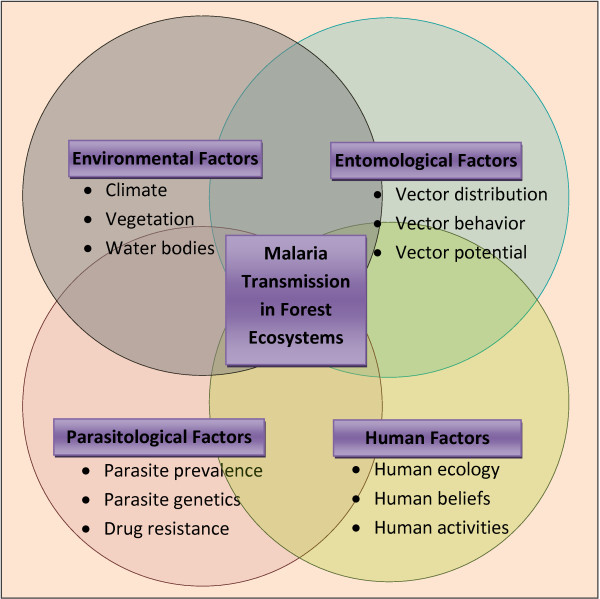
**Key factors influencing malaria transmission in forest ecosystems.** Situation specific malaria control strategies are warranted in forested areas based on in-depth understanding of the intricate relationships between key factors influencing malaria transmission dynamics.

Strong links exist between various factors influencing malaria transmission dynamics in forest ecosystems. Slight change in any of the factors affects the others, culminating in a different transmission pattern. Change in the vegetation cover and deforestation alters the distribution and behavior of malaria vectors. Human ecological and socioeconomic traits also affect exophagy, anthropophagy, biting rhythm, and resting behavior of vectors. The genetic traits of communities residing in forest areas and their health-seeking behaviors are crucial for parasite prevalence and precipitation of drug resistance. Moreover, insurgence of human populations and developmental activities in forests are important in altering the transmission pattern. Thus malaria transmission in forest areas is a complex process involving interplay between topographical, entomological, parasitological and human factors (Figure 1). Studies carried out in forested areas in different parts of the world generally focus on one or a few of the many factors which may not be adequate in understanding complexities of the malaria situation arising out of interaction of several factors. This review can help understand the complex interlinks between different factors acting simultaneously to influence malaria transmission. Predictive models of malaria transmission can be worked out for forest areas of different ecoregions by taking into account all relevant weighted factors. Based on in-depth understanding of the intricate relationship of various parameters, situation specific vector/malaria control strategies can be developed and implemented to address malaria problem in the forests. Although implementation of such strategies is primarily a responsibility of the government and local health authorities, NGO workers, local medicine practitioners and traditional faith healers would be important as these are acceptable to the communities residing in remote forested areas. At the same time establishing a good rapport through interaction between implementers and the communities is essential for the success and sustenance of malaria control programs in forest ecosystems.

## Competing interests

The authors declare that they have no competing interests.

## Authors’ contributions

NPK mined the literature as a part of his PhD thesis work. NPK and NN wrote the manuscript. AK, OP, and JMC added and improved overall manuscript structure and contents. All authors read and approved the final manuscript.
